# Supplementary figures and images for: Assessment of intra- and inter-genetic diversity in tetraploid and hexaploid wheat genotypes based on omega, gamma and alpha-gliadin profiles
Source: PeerJ. 2023 Nov 7;11:e16330. doi: 10.7717/peerj.16330 (PMC10637246; doi:10.7717/peerj.16330)

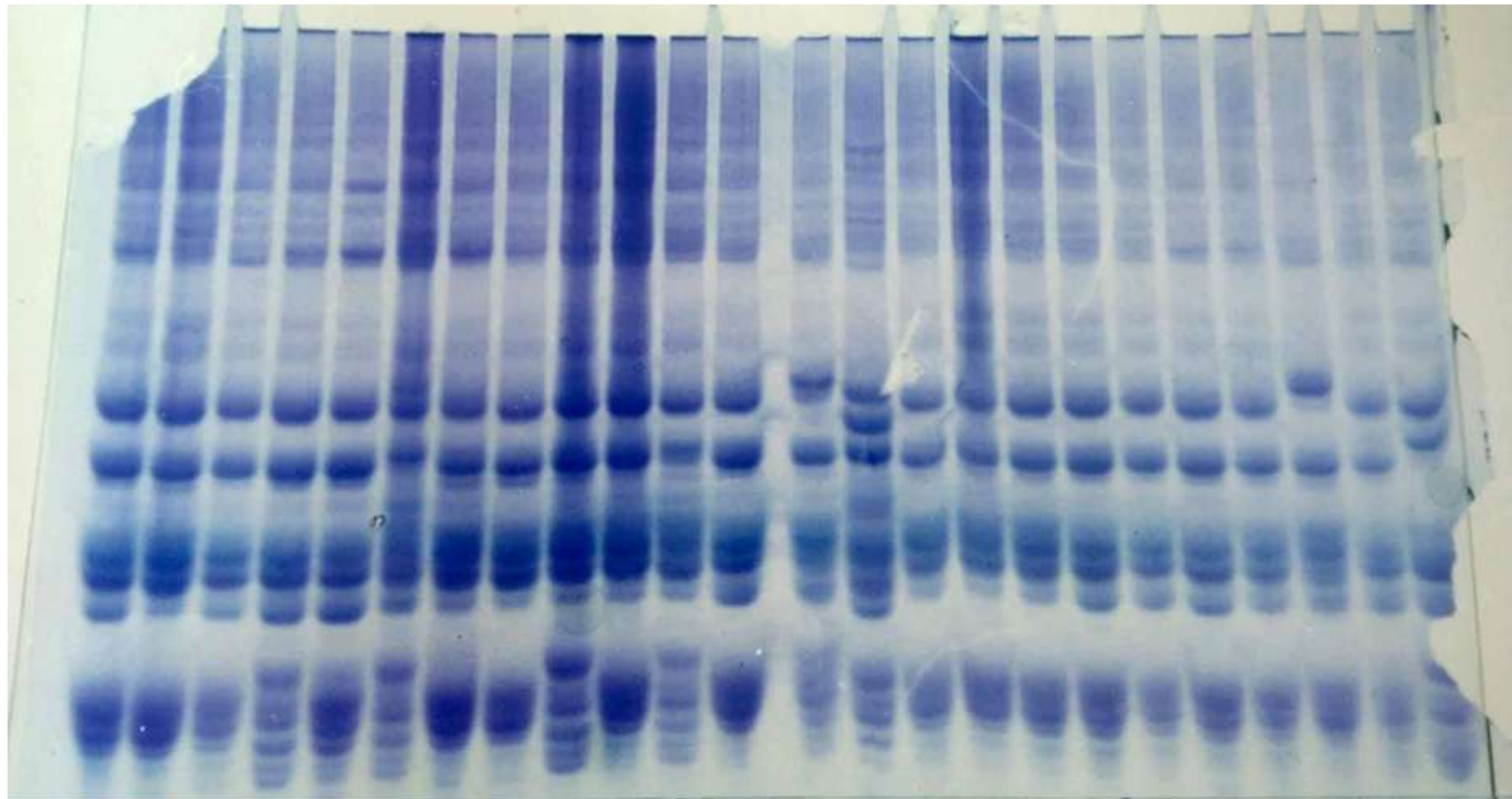

**Glutenine and gliadin banding patterns of 23 durum genotypes**

Supplement: Supplemental Information 1 [file peerj-11-16330-s001.pdf]

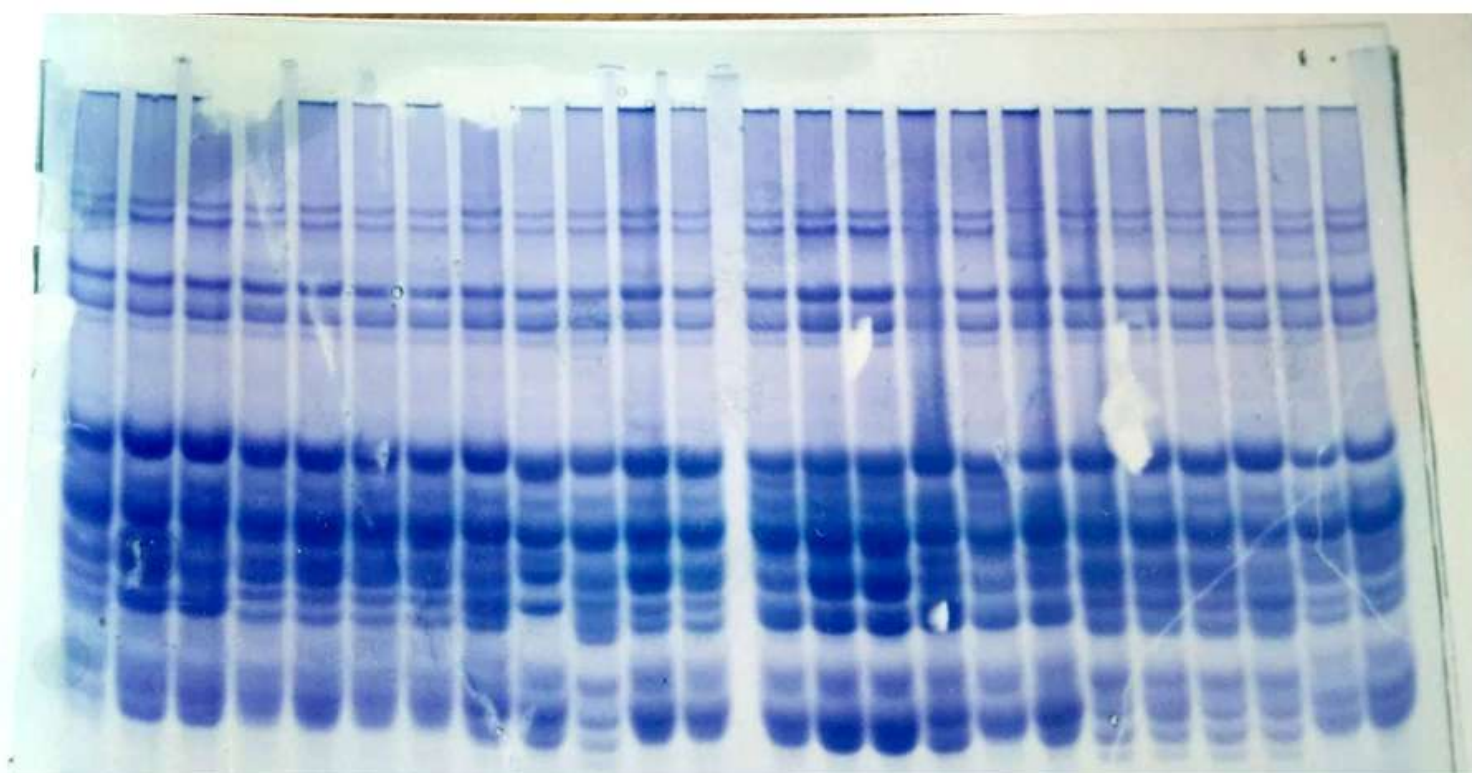

**Glutenine and gliadin banding patterns of 23 aestivum genotypes**

Supplement: Supplemental Information 2 [file peerj-11-16330-s002.pdf]
