# Supplementary material for: Assessment of intra- and inter-genetic diversity in tetraploid and hexaploid wheat genotypes based on omega, gamma and alpha-gliadin profiles
Source: PeerJ. 2023 Nov 7;11:e16330. doi: 10.7717/peerj.16330 (PMC10637246; doi:10.7717/peerj.16330)
Supplement: Supplemental Information 3 [file peerj-11-16330-s003.docx]

***Triticum turgidum***

>ADA67917.1 omega-gliadin [Triticum turgidum subsp. paleocolchicum]

MKTFLIFVLLAMAMKIATAARELNPSNKELQSPQQSFSHQQQPFPQQPYPQQPYPSQRPYPSQQPFPTPQ

QQFPQQSQQPFTQPQQPTPLQPQQPFPQQPQQPQQPFPQPQQPFPWQPQQPFPQTQQSFPLQPQQPFPQQ

PQQPFPQPQLPFPQQPEQIIPQQPEQIITQQPFPLQPQQAFPQQPQQPFPQPQQPIPVQPQQPFPQQSQQ

SQQPFPRPQQLFPELQQPIPQQAQQPFPQQSQQPFPQQPQQPFPLQPQQSFPQQPQQPFPQQPQQPFPLQ

PQQPFPLRPQQPFSQQPSILQPQQPQQPFLQPQQQLSQQLEQTISQQPHQPFPQQPHQPQQPYPQQQPSG

SSLTSIGGQ

>AAQ63861.1 gamma gliadin [Triticum turgidum subsp. durum]

MNIQVDPSSQVQWPQQQPFLQPHQPFSQQPQQIFPQPQQTFPHQPQQQFPQPQQPQRQFLQPRQPFPQQP

QQPYPQQPQQPFPQTQQPQQPFPQSKQPQQPFPQPQQPQQSFPQQQPSLIQQSLQQQLNPCKNFLLQQCK

PVSLVSSLWSIILPPSDCQVMRQQCCQQLAQIPQQLQCAAIHSVVHSIIMQQEQQEQLQGVQILVPLSQQ

QQVGQGILVQGQGIIQPQQPAQLEVIRSLVLQTLPTMCNVYVPPYCSTIRAPFASIVAGIGGQYR

>QDD55735.1 alpha gliadin [Triticum turgidum]

MKSFLILALLAIVATTATTAVRVPVPQLQPQNPSQQQPQEQVPLVQQQQFLGQQQQKFPGQQQPFPPQQP

YPQPQPFPPQLPFPQPQPFPPQQSYPQPQPQYPQPQQPISQQQAQQQQQQQQQQQQQQQQQQQQQQILQQ

ILQQQLIPCRDVVLQQPNIAHASSQVSQQSYQLLQQLCCQQLWQTPEQSRCQAIHNVVHAIILHQQQQQQ

QQQQQQPSSQVSYQQPQQQYPSAQGSFQPSQQNPQAQGFVQPQQLPQFEEIRNLALQTLPAMCNVYIPPY

CSTTIVPFGIIGTN

***Triticum turgidum***

>AJG03093.1 omega-gliadin [c]

MKTFIIFVLLAMAMNIASASRLLSPRDKELHTPQEQFPQQQQFPQPQQFPQQQIPQQHQIPQQPQQFPQQ

QQFLQQQQIPQQQIPQQHQIPQQPQQFPQQQFPQQQFPQQQFPQQEFPQQQQFPQQQIARQPQQLPQQQQ

IPQQPQQFPQQQQFPQQQSPQQRQFPQQQFPQQQQLPQKQFPQPQQIPQQQQIPQQPQQFPQQQFPQQQQ

FPQQQEFPQQQFPQQQFHQQQLPQQQFPQQQFPQQQFPQQQQFPQQQQLTQQQFPRPQQSPEQQQFPQQQ

FPQQPPQQFPQQQFPIPYPPQQSEEPSPYQQYPQQQPSGSDVISISGL

>AAQ63857.1 gamma gliadin [Triticum aestivum]

MNIQVDPSSQVQWPQQQPFLQPHQPFSQQPQQIFPQPQQTFPHQPQQQFSQPQQPQQQFIQPQQPFPQQP

QQPFPQTQQPQQPFPQSQQPQQPFPQPQQQFPQPQQPQQSFPQQQPSLIQQSLQQQLNPCKNFLLQQCKP

VSLVSSLWSMILPRSDCQVMRQQCCQQLAQIPQQLQCAAIHSIVHSIIMQQEQQEQRQGVQILVPLSQQQ

QVGQGILVQGQGIIQPQQPTQLEVIRSLVLQTLPTMCNVYVPPKCSIMRAPFASIVAGIGGQYR

>ABS72145.1 alpha gliadin [Triticum aestivum]

MKTFLILALLAIVATTATIAVRVPVPQLQPQNPSQQQPQEQVPLVQQQQFPGQQQPFPPQQPYPQPQPFP

SQQPYLQLQPFPQPQLPYPQPQLPYPQPQPFRPQQSYPQPQPQYSQPQQPISQQQQQQQQQQQQQILQQI

LQQQMIPCRDVVLQQHNIAYASSQVLQQSSYQLLQQLCCQQLWQIPEQSRRQAIHSVVHAIILHQQQQQQ

QQQQQQPSSQVSFQQPQQQYPSGQGSFQPSQQNPQAQGSLQPQQLPQFEEIRNLALQTLPAMCNVYIPPY

CTIAPFGIFGTN
